# Improving Delineation of True Tumor Volume With Multimodal MRI in a Rat Model of Brain Metastasis

**DOI:** 10.1016/j.ijrobp.2019.12.007

**Published:** 2020-04-01

**Authors:** James R. Larkin, Manon A. Simard, Axel de Bernardi, Vanessa A. Johanssen, Francisco Perez-Balderas, Nicola R. Sibson

**Affiliations:** Department of Oncology, Cancer Research UK and Medical Research Council Oxford Institute for Radiation Oncology, University of Oxford

## Abstract

**Purpose:**

Brain metastases are almost universally lethal with short median survival times. Despite this, they are often potentially curable, with therapy failing only because of local relapse. One key reason relapse occurs is because treatment planning did not delineate metastasis margins sufficiently or accurately, allowing residual tumor to regrow. The aim of this study was to determine the extent to which multimodal magnetic resonance imaging (MRI), with a simple and automated analysis pipeline, could improve upon current clinical practice of single-modality, independent-observer tumor delineation.

**Methods and Materials:**

We used a single rat model of brain metastasis (ENU1564 breast carcinoma cells in BD-IX rats), with and without radiation therapy. Multimodal MRI data were acquired using sequences either in current clinical use or in clinical trial and included postgadolinium T_1_-weighted images and maps of blood flow, blood volume, T_1_ and T_2_ relaxation times, and apparent diffusion coefficient.

**Results:**

In all cases, independent observers underestimated the true size of metastases from single-modality gadolinium-enhanced MRI (85 ± 36 μL vs 131 ± 40 μL histologic measurement), although multimodal MRI more accurately delineated tumor volume (132 ± 41 μL). Multimodal MRI offered increased sensitivity compared with independent observer for detecting metastasis (0.82 vs 0.61, respectively), with only a slight decrease in specificity (0.86 vs 0.98). Blood flow maps conferred the greatest improvements in margin detection for late-stage metastases after radiation therapy. Gadolinium-enhanced T_1_-weighted images conferred the greatest increase in accuracy of detection for smaller metastases.

**Conclusions:**

These findings suggest that multimodal MRI of brain metastases could significantly improve the visualization of brain metastasis margins, beyond current clinical practice, with the potential to decrease relapse rates and increase patient survival. This finding now needs validation in additional tumor models or clinical cohorts.

## Introduction

Brain metastasis is one of the most feared diagnoses for patients with cancer. Median survival for a patient with brain metastases is measured in months: around 7 months for the most treatable patients, dropping to 2 months in the worst cases.^1^^,^^2^ Before death, patients with brain metastases often experience seizures, paralysis, bleeding, behavioral disturbance, and headaches.

The conventional view of brain metastases is that they are discrete entities with a clear edge and a noninvasive phenotype. This model suggests that many brain metastases could be potentially cured by either surgical resection or radiation therapy. However, many patients have local relapse after therapy. Such relapse may reflect a number of problems, including surgical error, overly conservative treatment planning around eloquent areas of the brain, or radioresistance of the cells. However, it is becoming increasingly clear that planning simply may not always encompass the entire tumor volume because many metastases, even those that appear well circumscribed, actually have an invasive rim.^3^, ^4^, ^5^ Moreover, it is highly likely that this invasive rim is the reason why so many potentially curable patients have local relapse after therapy. An incomplete understanding of the tumor extent leads to flawed surgical or radiologic planning, incomplete treatment, and subsequent relapse. Despite this invasive rim, many brain metastases remain potentially curable if tumor extent can be more accurately delineated before treatment planning.

Currently, identification of the tumor rim relies upon independent observers interpreting imaging data, usually from single-modality gadolinium-enhanced magnetic resonance imaging (MRI). From these images, a gross tumor volume (GTV) is defined.^6^ Outside the GTV, margins of error are built in to account for nonvisible tumor before treatment planning. At present, the application of such margins remains the greatest unknown quantity in treatment planning. Attempts to accurately account for invasive margins have typically relied on historical postmortem series, meaning that margins are not individualized to patients. One reason why current imaging fails to capture tumor margins accurately may be that routinely used imaging sequences report on a relatively small number of properties. For example, they do not report on tumor-related functional parameters, such as blood flow and blood volume. Nevertheless, these parameters are critical aspects of tumor biology, and relevant data could be routinely acquired on clinical MRI systems.

The aim of this study, therefore, was to determine whether we can improve detection of the hidden tumor rim in brain metastases, both before and after radiation therapy treatment. Our hypothesis is that multimodal MRI, which reports on both structural and functional aspects of brain and tumor biology, can improve detection of the tumor margins that are missed by routine clinical diagnosis pathways.

## Methods and Materials

For all methods, see Methods E1 (available online at https://doi.org/10.1016/j.ijrobp.2019.12.007) for more details.

### Animal models

All animal experiments were approved by the UK Home Office (Animals [Scientific Procedures] Act 1986), conducted in accordance with the University of Oxford Policy on the Use of Animals in Scientific Research, the ARRIVE guidelines,^7^ and the Guidelines for the Welfare and Use of Animals in Cancer Research.^8^ To best recapitulate an isolated metastasis surrounded by normal brain, as is common in human patients, a model of brain metastasis induced through intracerebral injection was used. Although an intracerebral injection bypasses the initial stages of metastasis seeding, it recapitulates the later growth and expansion of macrometastases well.^9^ Systemic induction of metastases (eg, via intracarotid or intracardiac injection) yields a high number of very small metastases in the brain that are difficult to image and give rise to high morbidity before the metastases reach the macrometastatic stage.

**Fig. 1:**
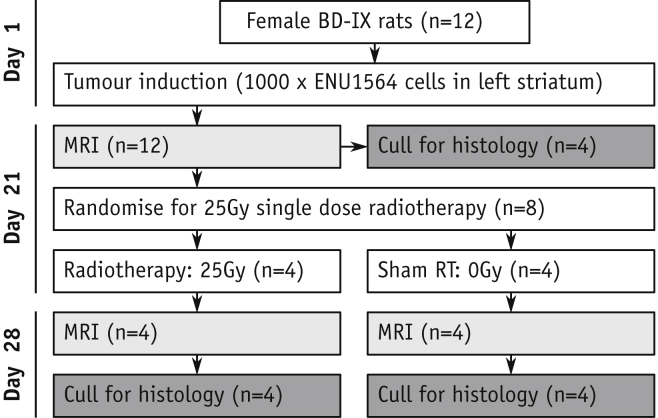
Study schematic indicating group structure. *Abbreviation*: MRI = magnetic resonance imaging.

Female Berlin-Druckery IX (BD-IX) rats (n = 12, 150-180 g) followed the flowchart in Figure 1. In brief, rats were injected with 1000 mycoplasma-free ENU1564 cells in the left striatum. The ENU1564 cells originated from an *N*-ethyl-*N*-nitrosourea-induced mammary carcinoma in a female BD-IX rat and readily metastasize to the brain. As such, this syngeneic cell line/rat strain model offers good biologic recapitulation of brain metastasis growth in a brain large enough for sufficiently high-resolution imaging, both of which are key requirements for this study. In a subset of animals (n = 4), the metastasis-bearing striatum was irradiated (25 Gy, single fraction, 225 kV x-rays; Fig. E1, available online at https://doi.org/10.1016/j.ijrobp.2019.12.007) using a SARRP irradiator (Xstrahl, Camberley, UK). Before euthanasia, animals received 60 mg/kg pimonidazole (Hypoxyprobe, Burlington, MA).

### MRI

MRI was conducted at 9.4T (Agilent Technologies Inc, Santa Clara, CA) with a 72 mm volume-transmit coil and a 4-channel surface-receive array (Rapid Biomedical, Rimpar, Germany). In each MRI session, baseline maps for T_1_ and T_2_ relaxation times (seconds), cerebral blood flow (CBF; mL/100g/min) using arterial spin labeling (ASL),^10^ cerebral blood volume (CBV; %) using blood-pool labeling with iron oxide nanoparticles,^11^ and apparent diffusion coefficient (ADC; μm^2^/ms) were acquired (spin-echo echo planar imaging readout; field of view = 32 × 32 mm, matrix = 64 × 64, thickness = 1 mm). Structural T_2_-weighted and pre- and postgadolinium (Gd) T_1_-weighted images were also acquired (field of view = 32 × 32 mm, matrix = 256 × 256, thickness = 1 mm).

### Histology

Three interleaved sets of 20 μm sections were taken every 200 μm and stained to identify tumor cells (epithelial cell adhesion molecule; EpCAM), hypoxia (pimonidazole), or vessels (collagen IV). All were counterstained with cresyl violet. Scanned images were deconvolved into brown (DAB; EpCAM, pimonidazole or vessels) and blue (cresyl violet; nuclei) using ImageJ and were binary-thresholded to produce masks of stained areas using a uniform threshold value for all images of each type. Masks were downscaled without interpolation to 2 μm/pixel. Pimonidazole, cresyl violet, and EpCAM stains were processed to yield maps of percentage staining by uniform threshold. Vessel binary masks were preprocessed to remove entities <5 pixel (noise) or >4000 pixel (ventricles/staining artifacts). Vessel masks were processed with in-house Matlab (MathWorks, Natick, MA) functions to produce parameter maps of vessel area fraction, central diameter, cross-sectional area, density, and in-plane length (code available upon request). All histologic images and maps were combined and transformed into MRI-space volumes. Full details of histology transformation and combination methods are given in the Supplementary Information (available online at https://doi.org/10.1016/j.ijrobp.2019.12.007). In brief, histology images were matched to MRI slices based upon anatomy and with reference to a brain atlas,^12^ underwent perspective transforms to account for tissue distortion during sectioning, and were masked to account for damaged or missing tissue. Final MRI-space slices were produced by averaging all contributing histologic sections (typically 5 sections, each 200 μm apart) and converting spatial coordinates so that the histologic volume matched the MRI slice. Multiple MRI-space slices were combined to yield each complete MRI-space volume (see Fig. E2 and E3 for example section images at each stage and histology parameter images; available online at https://doi.org/10.1016/j.ijrobp.2019.12.007).

### Tumor extent maps

Four independent observers each delineated maximum tumor extent on postgadolinium T_1_-weighted images. A single independent-observer consensus was produced by excluding voxels for which only a single observer had defined tumor.

To assess whether automated analysis of the T_1_-weighted post-Gd images offered improvement over independent observers, 2 methods of identifying tumors from post-Gd T_1_-weighted images were used: (1) voxels >2 standard deviations (SDs) above the mean signal intensity of the contralateral (normal) hemisphere and (2) voxels defined by an automated routine designed to more accurately emulate steps a human intuitively makes (termed “refined” method; for exact step methodology see Supplementary Information; available online at https://doi.org/10.1016/j.ijrobp.2019.12.007).

Gold-standard tumor maps were produced for each animal using the MRI-space transformed volumes of histologic data to minimize variations arising from differing resolutions. Voxels were considered as tumor if they were either >0.001% EpCAM-staining positive or >1% pimonidazole-staining positive. These thresholds were chosen from manual observation of contralateral staining intensity and calculations of expected staining intensity at a given tumor cell density in the extreme periphery of the tumor (see justification in Fig. E4; available online at https://doi.org/10.1016/j.ijrobp.2019.12.007). Multimodal MRI tumor maps were produced by considering voxels that were abnormal for 2 or more parameters as tumor. A voxel was considered abnormal if it was >1 SD above the contralateral mean for post-Gd T_1_-weighted image intensity, baseline T_1_ time, or ADC, or if it was >1 SD below the contralateral mean for baseline T_2_ time, CBV, CBF, or ADC (Table 1). See Supplementary Information for additional details and a full justification of this method (available online at https://doi.org/10.1016/j.ijrobp.2019.12.007). In brief, combining 2 parameters, each 1 SD from the mean, minimizes false positives by keeping α < 0.05.

**Table 1:** Imaging parameters and threshold values considered “abnormal”

Imaging parameter	Units	Threshold for inclusion
Post-Gd T_1_-weighted image intensity	Arbitrary units	>1 SD above contralateral mean
Baseline T_1_ time	Seconds	>1 SD above contralateral mean
Baseline T_2_ time	Seconds	>1 SD below contralateral mean
Cerebral blood volume	Percentage	>1 SD below contralateral mean
Cerebral blood flow	mL/100 g/min	>1 SD below contralateral mean
Apparent diffusion coefficient	μm^2^/ms	>1 SD above or below contralateral mean

*Abbreviations*: Gd = gadolinium; SD = standard deviation.

The contralateral hemisphere, for both MRI and histology, was defined as any region of the brain beyond the midline on the side of the brain without tumor, excluding ventricles (defined by T_2_-weighted imaging) and accounting for any tumor-induced midline shift. This contralateral region was not subdivided further (eg, gray or white matter regions) because doing so would either limit contralateral region size or introduce unacceptable errors owing to partial volume effects at this resolution.

Sensitivities, specificities, and accuracies for identifying tumor on a voxel-wise basis were determined for each method using histologically identified tumor as the gold standard. The importance of each MRI modality for accurate tumor delineation was determined by systematic removal of each modality in turn and recalculation of sensitivities and specificities. The importance of each modality for finding occult tumor rim (ie, tumor confirmed histologically and missed by independent observers) was determined by eliminating all voxels identified by expert observers and recalculating sensitivities and specificities.

### Statistical analysis

Unless otherwise specified, differences between groups were determined by analysis of variance followed by Tukey or Sidak multiple comparison post hoc test. Nonparametric comparisons used Wilcoxon signed-rank test. Comparisons between classification methods were made using matched sample contingency tables and McNemar test.^13^ Group-wise analysis of regions of interest (ROIs) from individual animals used random-effects weighted mean models.^14^

## Results

### Tumour appearance, size, and effect of radiotherapy

**Fig. 2:**
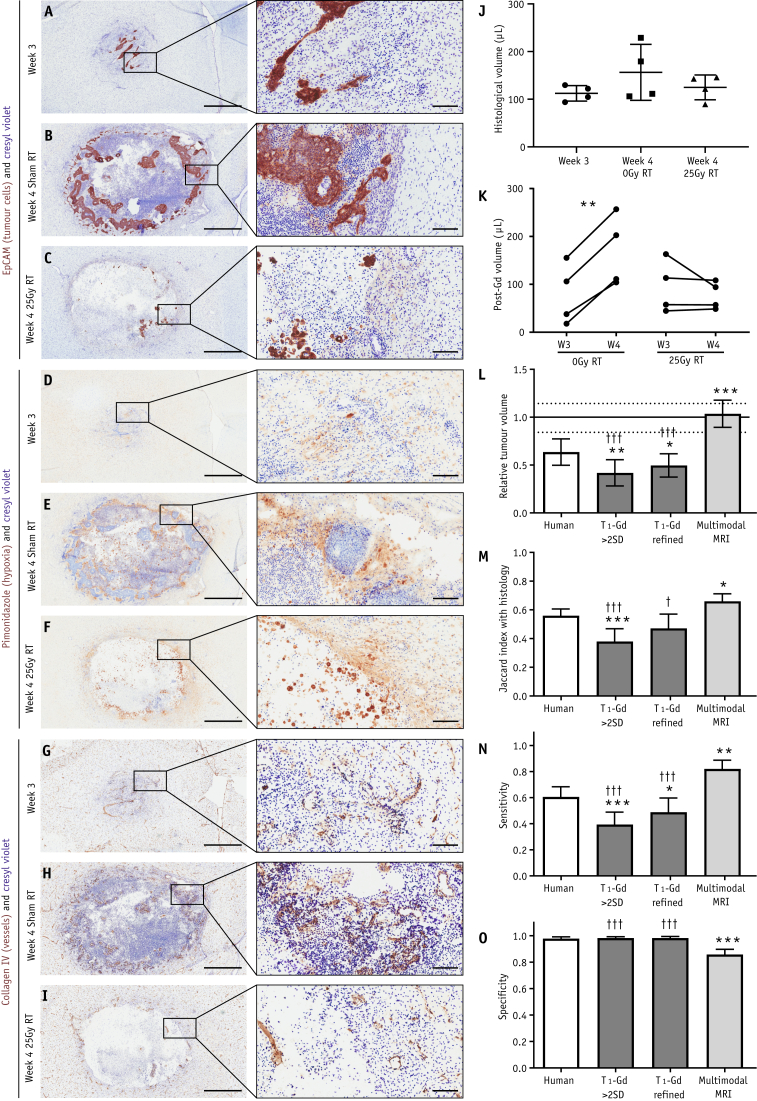
Tumor appearance, size, and detection by manual and automated methods. (A-I) Photomicrographs of tumor showing brown EpCAM staining (tumor; A-C), pimonidazole staining (hypoxia; D-G) or collagen IV staining (vessels; G-I), each with blue cresyl violet counterstain at week 3 (A, D, and G), week 4 after sham radiation therapy (RT; B, E, and H), and week 4 after 25 Gy radiation therapy (C, F, and I). Photomicrographs are from adjacent 20 μm sections from the same animals. Scale bars are 1 mm for widefield panels and 100 μm for magnified insets. (J) Histologic tumor volume in each animal. (K) Pre- and post-radiation therapy tumor volumes, as assessed by independent observation of post-Gd T_1_-weighted magnetic resonance imaging (MRI) data sets. (L) Tumor volume detected by each method relative to the mean histologic size within each of the 3 groups of animals; mean ± 95% confidence intervals (CI). Mean and 95% CI for histologic detection are solid and dashed horizontal lines, respectively; n = 12 in each group. (M) Jaccard indices between tumor maps produced by each of the 4 methods and histologic gold-standard tumor maps; n = 12 in each group. (N) Sensitivity and (O) specificity of each method for detecting voxels identified as tumor histologically; data are given as mean + 95% CI; n = 12 in each group. *P < .05, **P < .01, ***P < .001 relative to human observer; †*P* < .05, †††*P* < .001 relative to multimodal MRI, analysis of variance followed by Sidak multiple comparison test.

Tumors at week 3 were largely uniform with increased cellular density. As tumors grew, hypoxia and cellular density increased, pockets of necrosis became evident, and an infiltrative edge developed. After radiation therapy, a necrotic cyst formed, leaving a less distinct rim of cells surrounding it (Fig. 2A-2I). Histologic tumor volume was 112 ± 16 μL, 156 ± 59 μL, and 125 ± 26 μL at week 3, week 4 after sham (0 Gy) radiation therapy, and week 4 after 25 Gy radiation therapy (Fig. 2J), respectively. As monitored by independent-observer consensus using T_1_-weighted post-Gd images, tumors grew between weeks 3 and 4 in animals receiving sham radiation therapy (79 ± 63 μL to 170 ± 74 μL, n = 4, *P* < .01, paired *t* test), but not in animals receiving radiation therapy (95 ± 55 μL to 77 ± 29 μL, n = 4, Fig. 2K). Tumor volume, as measured from T_1_-weighted post-Gd images for all animals at week 3 was 94 ± 50 μL (n = 12) and was not significantly different between groups of animals after randomization for radiation therapy, sham radiation therapy, or histology (n = 4 each).

Contralateral ROIs were defined for each animal (mean volume 156 ± 40 μL) and normal histologic parameters calculated in a pooled contralateral ROI from all animals (Table E1; available online at https://doi.org/10.1016/j.ijrobp.2019.12.007). Voxels with missing histologic data were excluded from all analyses. Histology-based tumor maps were considered the gold standard.

### Comparison of methods for tumor delineation

Independent-observer agreement was similar to previously published multicenter analyses.^15^ Intrabrain, interobserver tumor volume error was 14% ± 7%. Tumor overlap between observers (Jaccard Index, *J*), was 0.76 ± 0.06. Interobserver variation in *J* was 6.1% ± 4%. For additional analyses, see Supplementary Information (available online at https://doi.org/10.1016/j.ijrobp.2019.12.007). Tumor volumes from “>2 SD” and “refined” automated methods were smaller than those drawn by independent observers (Fig. 2L). Both automated methods showed poorer spatial overlap with histology than both independent-observer analysis and multimodal MRI analysis (Jaccard index; Fig. 2M). Sensitivities for the >2 SD and refined methods were 0.39 ± 0.15 and 0.49 ± 0.17 (35% and 19% lower than independent observer analysis, respectively; *P* < .001), and 52% and 40% lower than the multimodal MRI analysis (*P* < .001; Fig. 2N), although specificities were unchanged (Fig. 2O).

**Fig. 3:**
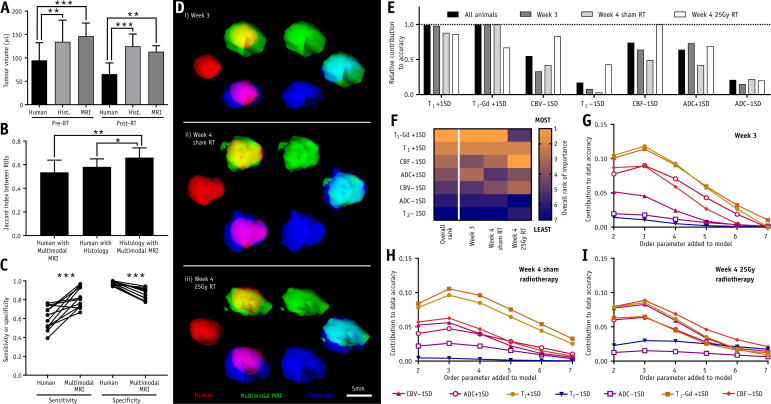
Tumor volumes, overlaps, and prediction results. (A) Tumor volumes for groups of animals that have untreated tumors pre–radiation therapy (pre-RT; n = 8) and animals that have radiation therapy-treated tumors (post-RT; n = 4). Volumes are determined by consensus of independent observers, histologic gold standard, or multimodal magnetic resonance imaging (MRI) analysis (**P < .01; ***P < .001; repeated-measures analysis of variance with Tukey’s post hoc test). (B) Jaccard indices indicating extent of overlap between each pair of maps produced by different methods (n = 12 per group; *P < .05; **P < .01; Friedman test with Dunn’s post hoc comparisons). (C) Sensitivity and specificity for detecting tumor-containing voxels in each animal by either human observer or multimodal MRI (***P < .001; paired *t* tests). (D) Three-dimensional renderings of human observer (red), multimodal MRI (green), and histologic gold-standard (blue) tumor volumes, together with overlays of each pair of volumes, for an example animal from each group. (E) Relative contribution to model accuracy for each MRI abnormality included in the classification model. (F) Heatmap showing ranking for each modality, both in all animals combined (overall rank) and in tumors from the 3 individual groups. Rankings run from most important (1) to least important (7). (G-I) Absolute contributions for each modality to model accuracy for tumor delineation (delta accuracy) in tumor-bearing animals at (G) week 3, (H) week 4 after sham radiation therapy, and (I) week 4 after 25 Gy radiation therapy. Contributions are shown for the order of adding each modality to the classification models. Higher delta accuracy, especially at lower order numbers, indicates greater importance for the modality in accurate tumor delineation. See Fig. E5 (available online at https://doi.org/10.1016/j.ijrobp.2019.12.007) for example interpretations. *Abbreviations*: ADC = apparent diffusion coefficient; CBF = cerebral blood flow; CBV = cerebral blood volume.

Independent observers drew smaller tumor volumes in both pre- and post-radiation therapy groups than were found by either histology or multimodal MRI (pre-radiation therapy: independent observer at 95 ± 38 μL vs histology at 134 ± 46 μL [*P* < .05], or multimodal MRI at 146 ± 28 μL [*P* < .001]; post-radiation therapy: independent observer at 65 ± 24 μL vs histology at 124 ± 26 μL [*P* < .01], or multimodal MRI at 113 ± 13 μL [*P* < .01]; Fig. 3A). Considering volume coregistration, J_human-histology_ and J_human-multimodal_ were both lower than J_histology-multimodal_ (*P* < .05 and *P* < .01, respectively), indicating better agreement between multimodal MRI and histology than between independent-observer analysis and histology (Fig. 3B).

Considering all tumors, sensitivity and specificity for independent observers to identify histologically confirmed tumors were 0.61 (95% confidence interval [CI], 0.53-0.68) and 0.98 (95% CI, 0.96-0.99), respectively. Sensitivity and specificity were not different between groups of animals. Multimodal MRI increased sensitivity to 0.82 (95% CI, 0.75-0.89, *P* < .001), but decreased specificity to 0.86 (95% CI, 0.82-0.90, *P* < .001; Fig. 2N-2O and 3C). The Youden index (Y=Sensitivity+Specificity−1) was higher for multimodal MRI (0.68) than for independent observers (0.59), indicating net benefit in delineating tumors for multimodal MRI. Example 3-dimensional renderings of delineated tumor volumes from each group are shown in Fig. 3D.

### Modality importance

Overall, using 2 abnormal MRI modalities to identify tumor was optimal (see full justification in Supplementary Information; available online at https://doi.org/10.1016/j.ijrobp.2019.12.007). However, modalities did not contribute equally to tumor identification, with some modalities offering greater improvements in accuracy than others. Relative contribution of different modalities to overall model accuracy, as well as breakdowns for each group, are given in Figure 3E. The 3 most important modalities at week 3 were, in order, T_1_ post-Gd intensity, baseline T_1_ time, and increased ADC; for week 4 tumors after sham irradiation, decreased CBF replaced increased ADC as the third most important metric. In contrast, for week 4 tumors after 25 Gy irradiation, the top 3 modalities were CBF, baseline T_1_ time, and CBV. A heatmap of modality rankings is shown in Figure 3F.

Although in a complete classification model the order of addition of MRI modalities does not affect the outcome, there may be advantages in a clinical setting to limiting the length of acquisitions. In such cases, fewer modalities will be acquired. Consequently, the increase in absolute model accuracy offered as each modality is added in a particular order is important because it will inform acquisition protocols and enable these to be limited, where necessary, to those modalities that will contribute the most. Absolute MRI modality accuracy contributions varied between groups and was dependent on the addition order (Fig. 3G-3I; Fig. E5, available online at https://doi.org/10.1016/j.ijrobp.2019.12.007). For week 3, accuracy varied depending on which parameters were added to the analysis early (first to fourth additions), shown by wide modality dispersions in the contribution to data accuracy (delta accuracy) plots (Fig. 3G); dispersion decreased as modalities were added. Combined with the intercept at ∼0 delta accuracy for addition 7, this finding indicates that tumors are mostly delineated using a few key modalities, mainly baseline T_1_ time, T_1_ post-Gd intensity, ADC, and CBF (Fig. 3G). Week 4 tumors, with or without radiation therapy, had lower accuracy variance when modalities were added early and higher delta accuracy for the seventh addition than week 3 tumors (F-test, *P* < .001, paired *t* test, *P* < .05, respectively; Fig. 3H-3I). Combined, these data show that more modalities are needed for high accuracy in larger, heterogeneous tumors.

**Fig. 4:**
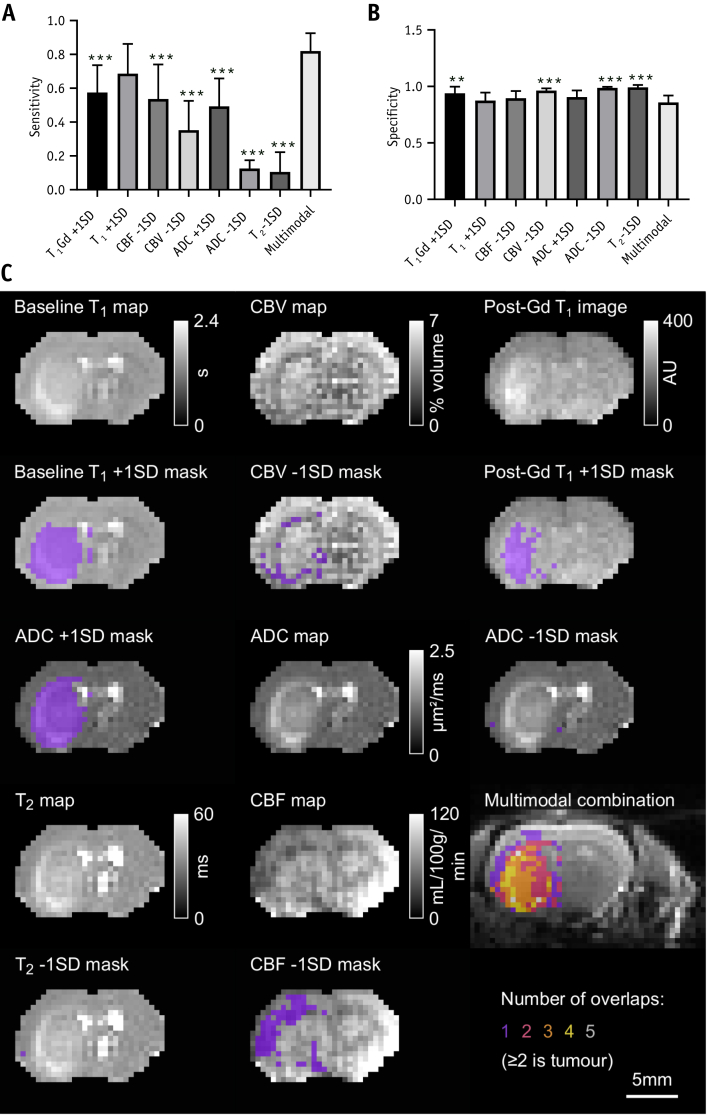
Comparisons with individual modalities and example MRI scans. (A) Sensitivity and (B) specificity of tumor detection using individual MRI metrics, as well as the multimodal MRI method. **P < .01; ***P < .001 with respect to multimodal MRI; analysis of variance followed by Dunnet’s post hoc test. (C) Example MRI maps and images, alongside overlays of the same map or image with the relevant threshold mask. The multimodal combination map shows the final spatial localization of the tumor by the multimodal MRI method (the multimodal MRI tumor definition is the area where at least 2 modalities have identified an abnormality). *Abbreviations*: ADC = apparent diffusion coefficient; CBF = cerebral blood flow; CBV = cerebral blood volume; MRI = magnetic resonance imaging.

Individually, no modality was as effective as the multimodality combination. Sensitivities for tumor detection were lower for every modality, except baseline T_1_ time, than for the multimodal MRI (*P* < .001); sensitivity for individual metrics ranged from 0.11 (95% CI, 0.03-0.18; T_2_ − 1 SD) to 0.69 (95% CI, 0.57-0.80; T_1_ + 1 SD), compared with 0.82 (95% CI, 0.75-0.89) for multimodal MRI (Fig. 4A). Specificities for single-modality tumor detection were often higher than multimodal MRI (Fig. 4B), reflecting the smaller areas delineated by these individual metrics. Example maps showing MRI data, masks, and a demonstration of how the combination of modalities is more effective than the individual metrics are given in Figure 4C.

### Occult tumor detection and characterization

**Fig. 5:**
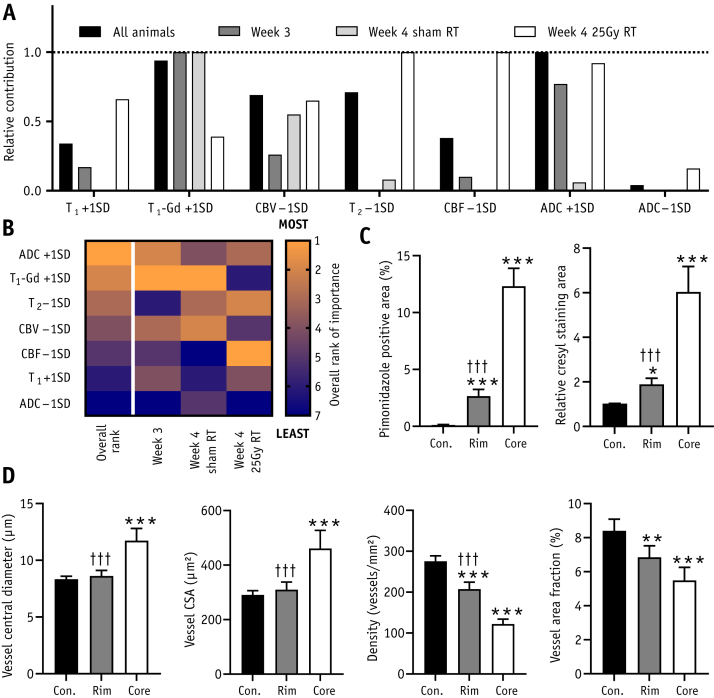
Characterizing clinically occult tumor rim. (A) Relative contribution to model accuracy for each magnetic resonance imaging abnormality when assessing the identification of the clinically occult tumor rim only (ie, histologically validated tumor, missed by independent observers). (B) Heatmap showing ranking for each modality, both in all animals combined (overall rank) and in tumor rims from the 3 individual groups. Rankings run from most important (1) to least important (7). (C, D) Histologic property comparisons for pimonidazole (hypoxia) and cresyl violet (cellularity) staining (C) and vessel parameters (D). Comparisons are made between (i) the normal contralateral hemisphere (Con.), (ii) the tumor core identified by independent observers (core), and (iii) the tumor rim, beyond the core, and identified by multimodal magnetic resonance imaging but not independent observer (Rim). n = 12 per group; *P < .05, **P < .01, ***P < .001 relative to contralateral hemisphere; †††*P* < .001 with respect to tumor core. Group-wise random effects weighted mean models followed by analysis of variance with Tukey’s post hoc test. *Abbreviations*: ADC = apparent diffusion coefficient; CBF = cerebral blood flow; CBV = cerebral blood volume; RT = radiation therapy.

We defined “clinically occult tumor rim” as histologically confirmed tumor that was not identified as tumor on single-modality post-Gd T_1_-weighted MRI by independent observers. This tumor region, although missed by human observation, nevertheless contains regions with abnormal MRI metrics. Thus, our multimodal MRI approach identifies tumor in these “occult” rims, which human observers miss. Considering just this clinically occult tumor rim, the overall relative contributions to multimodal MRI model accuracy for each modality across all animals are given in Figure 5A, with a heatmap of ranking given in Figure 5B. The most important overall metrics for identifying this rim were ADC, T_1_ post-Gd, and T_2_. The patterns of relative contribution to accuracy for each modality were different in the rim alone (Fig. 5A, 5B), compared with the entire tumor (Fig. 3E, 3F).

Histologically, the additional region of tumor found by multimodal MRI is still markedly hypoxic and shows increased cellularity relative to normal brain (Fig. 5C). Analysis of rim vessel structure identified abnormalities relative to normal brain, including increased vessel diameter and decreases in vessel density and overall vessel area fraction (Fig. 5D). For many histologic properties, the rim was abnormal relative to normal brain but represented a less extreme divergence from normal tissue properties than the tumor core. A complete summary of normal histologic parameters is given in Table E1 (available online at https://doi.org/10.1016/j.ijrobp.2019.12.007).

## Discussion

Delineating the full extent of the infiltrative tumor edge is critical for successful therapy planning. Up to 60% of patients undergoing brain metastasis therapy relapse locally,^16^, ^17^, ^18^ and it is highly likely that a fraction of these relapses are owing to underestimation of the extent to which metastases infiltrate into normal-appearing brain. Here, we have combined several MRI modalities to improve detection of the infiltrating tumor margin in a single rat model of breast cancer brain metastases. Each modality has been chosen for being both relatively simple to implement and able to reveal information on properties of tissue that are likely to be modified in a tumor, from cellularity to blood flow. We have shown that it is possible to combine these modalities in a simple protocol to objectively and more accurately define brain metastasis margins compared with either independent-observer assessment or single-modality automated analysis. The multimodal MRI approach had higher spatial agreement with histology, as measured by the Jaccard index, than any of the individual MRI metrics or the independent observers. Independent observers using post-Gd T_1_-weighted MRI underestimated both the size and the extent of metastases, whereas multimodal MRI estimates were in good agreement with histology. All MRI parameters acquired contributed to metastasis volume definition, but those contributing the most varied depending on metastasis pathology. Traditional metrics, including post-Gd T_1_-weighted imaging, were more important for untreated metastases, whereas blood flow/volume-based metrics were more important for post-radiation therapy metastases, as well as identification of the metastasis rim that was identified histologically and by multimodal MRI but missed by independent observers on single-modality post-Gd MRI.

Current clinical methods for defining tumor volumes are highly subjective. Disagreement has been reported previously between observers for identical metastases with MRI,^15^ CT,^19^ and radiosurgery planning,^20^ and this was corroborated in the present study. Alternative proposals for determining margins include magnetic resonance spectroscopy imaging,^21^^,^^22^ multimodal nanoparticles,^23^ or positron emission tomography/MRI.^24^ However, each has drawbacks, including poor resolution, licensing requirements, and cost.

One key finding herein is that disrupted perfusion is particularly important in defining metastasis margins in a post-radiation therapy setting. Although blood-based parameters, flow in particular, have been shown to be useful for diagnosis and classification of brain tumors,^25^^,^^26^ neither study sought to identify the invasive edge of tumors. For the invasive edge, one previous report has suggested that CBV is more important than spectroscopy for determining glioma periphery.^27^

Clinical success for our approach requires multiple MRI pulse sequences. Although the most important modality is the routinely acquired post-Gd T_1_-weighted imaging, this does not hold true in all tumors and is insufficient for accurate margin identification without other modalities. However, it is noteworthy that in the multimodal analysis the T_1_ post-Gd relative contribution was still high in the occult rim missed by independent observers, indicating that an element of human fallibility contributes to the lack of accurate rim identification. Nevertheless, the additional modalities used in this study are routinely acquired, available on the majority of scanners, or very close to wide clinical implementation. Sequences for mapping ADC, T_1_, and T_2_ relaxation times are built into most clinical scanners. Although we acquired our CBV mapping through injection of ultrasmall superparamagnetic particles of iron oxide, which is not routinely performed in the clinic, it is the final map that is important, not the method of acquisition. This means that dynamic susceptibility contrast data, acquired during gadolinium administration and which can be used to generate CBV maps,^28^ can substitute for ultrasmall superparamagnetic particles of iron oxide infusion. For CBF, the preferred technique is ASL, requiring no exogenous agents and producing quantitative maps in short times. ASL has been used in more than 200 trials and has clinical acquisition guidelines^29^; consequently, it is a matter of when, not if, vendors will include the pulse sequence routinely. Moreover, only a subset of scans may be required, and this may be preferable in a busy clinical setting. Our results on the order of addition (Fig. 3G-3I) can be used in this case to determine the priority of acquisition in terms of increased model accuracy gained by adding modalities to the scan protocol. Finally, because only relative quantitative data are required, the potential for immediate clinical translation across multiple centers is high and minor differences between centers can be resolved using in-patient normalization; this will be an important point for optimization in a clinical implementation. In this study, we used a single contralateral reference region including both gray and white matter. In doing so, we have increased the standard deviation of parameters and made the approach less sensitive than it might have been had local determination of normal parameters been possible. The higher relative resolution (ie, voxel size relative to brain size) available in clinical systems will mean that such approaches may offer increased utility in patients. We ultimately envisage a clinical pathway where coregistered image sets are input into a simple program with a defined normal brain region. The output map will provide the GTV, which will then be enlarged to a clinical treatment volume during radiologic or surgical planning. Thus, the improvement that this methodology offers is a more accurate representation of the actual tumor volume in the GTV than is possible currently, both for untreated and pretreated tumors (eg in an imaging session part-way through a fractionated radiation therapy course).

## Conclusions

Tumor invasion into normal brain is one overwhelming reason why local therapies fail and brain metastases relapse. It is essential, therefore, that we find better ways to accurately delineate this invasive margin. Here, we have provided strong evidence that simple combination of multimodality MRI may be able to delineate the true metastasis extent better than current clinical practice. This approach is objective, and the required acquisitions are either clinically available or are moving toward routine use in the near future. Coupled with a simple analysis pipeline, there is a ready path for translation, and validation studies of this approach in other animal models or patient populations are now warranted.
