# Supplementary material for: Improving Delineation of True Tumor Volume With Multimodal MRI in a Rat Model of Brain Metastasis
Source: Int J Radiat Oncol Biol Phys. 2020 Apr 1;106(5):1028–38. doi: 10.1016/j.ijrobp.2019.12.007 (PMC7082766; doi:10.1016/j.ijrobp.2019.12.007)
Supplement: Supplementary Information [file mmc1.pdf]

Supplementary information to accompany:

Improving delineation of true tumour volume with multimodal MRI in a rat model of brain metastasis

James R. Larkin, Manon A. Simard, Axel de Bernardi, Vanessa A. Johanssen, Francisco Perez-Balderas, Nicola R. Sibson

Available from https://doi.org/10.1016/j.ijrobp.2019.12.006

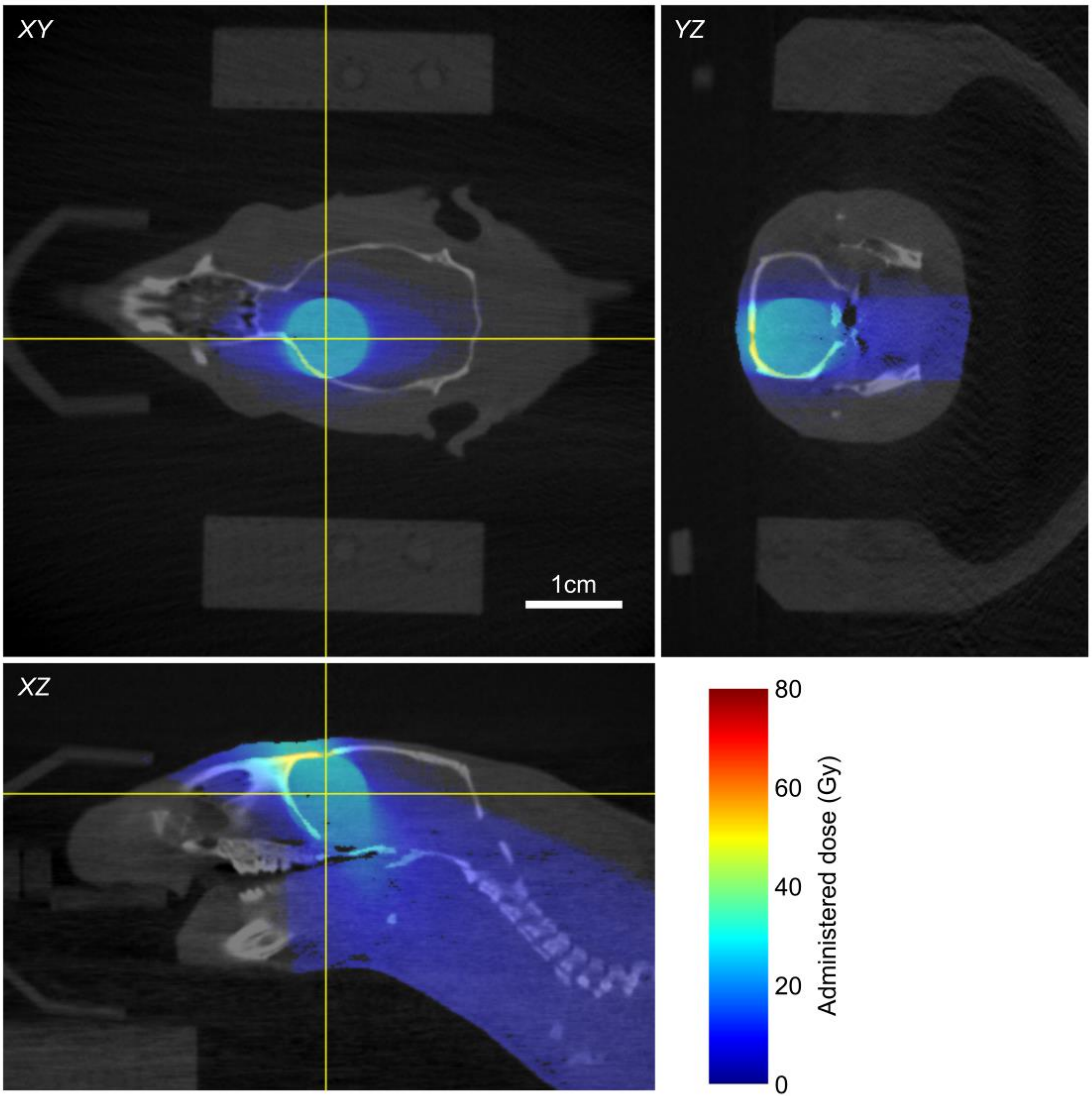

Figure E1: Example dose pattern. Example radiation dose pattern administered to rats in the study. Collimator gave a circular beam 8mm in diameter at the isocentre. Target dose was 25Gy using 225kV X-rays. The beam pattern was a 70° arc along the transverse plane of the rat from a point directly above the tumour to a point anterior to the rat (designed to spare the contralateral hemisphere and minimize non-tumour irradiation). Tumour isocentre was positioned by using T_1-weighted post-Gd MRI to determine isocentre relative to the skull midline (*XZ* plane), top of the cortex (*XY* plane) and the olfactory sulcus (*YZ* plane), before then transferring these relative coordinates to the CT of the head for irradiation.

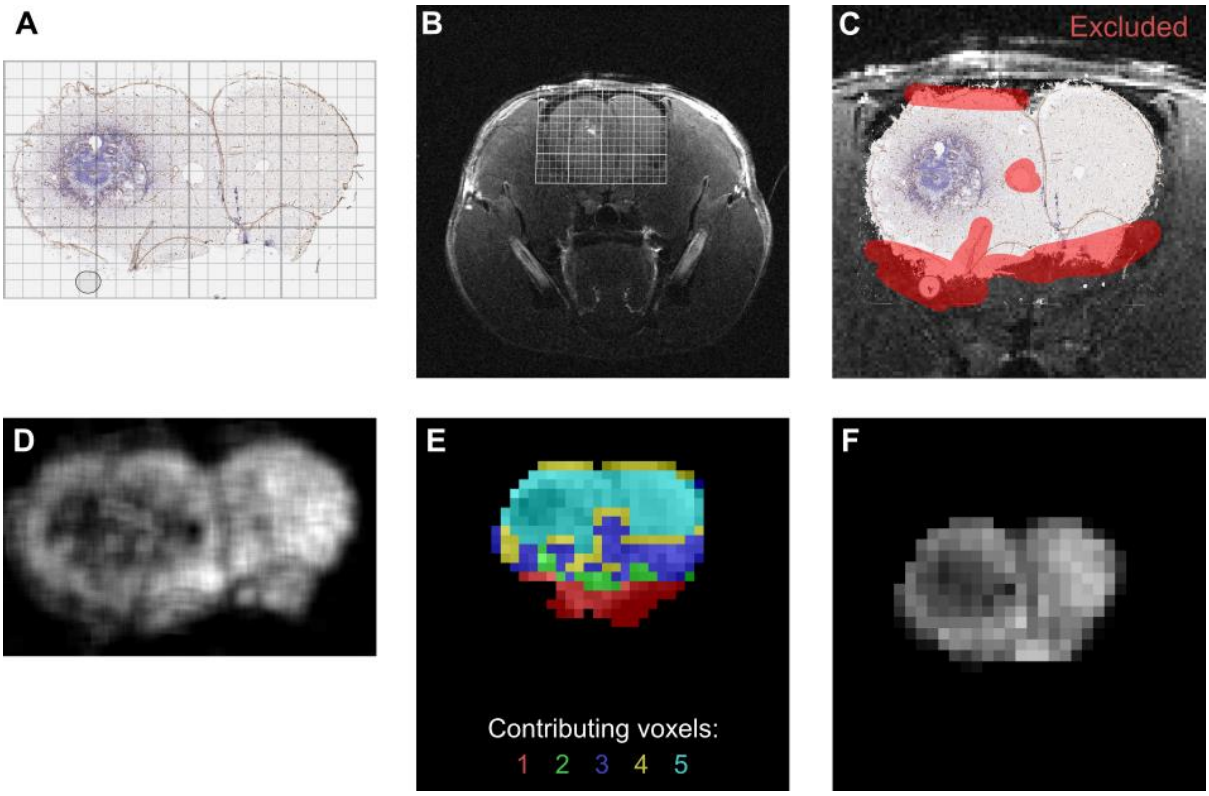

Figure E2: Perspective transform and histological combination to MRI-space (A) Original histological image with overlay grid before perspective transform. (B) Representation of manually-determined perspective transformation matrix determined for this histology section to allow overlay of histology with MR image. (C) Poor quality tissue, e.g. tears or other artefacts, is masked for exclusion. (D) Parameter maps are created from original image on a rolling average basis to allow immediate downscaling to MR space, irrespective of transformation. Each pixel represents the mean value for a square 0.5mm on each side, centred on the pixel. (E) Multiple histological sections for each MR slice are averaged, taking into account tissue marked for removal; colour code indicates number of histological sections that contribute to each voxel after exclusions. (F) Final MR-space maps of histological parameters are produced by combining multiple MR-resolution slices, each prepared from up to five histological sections.

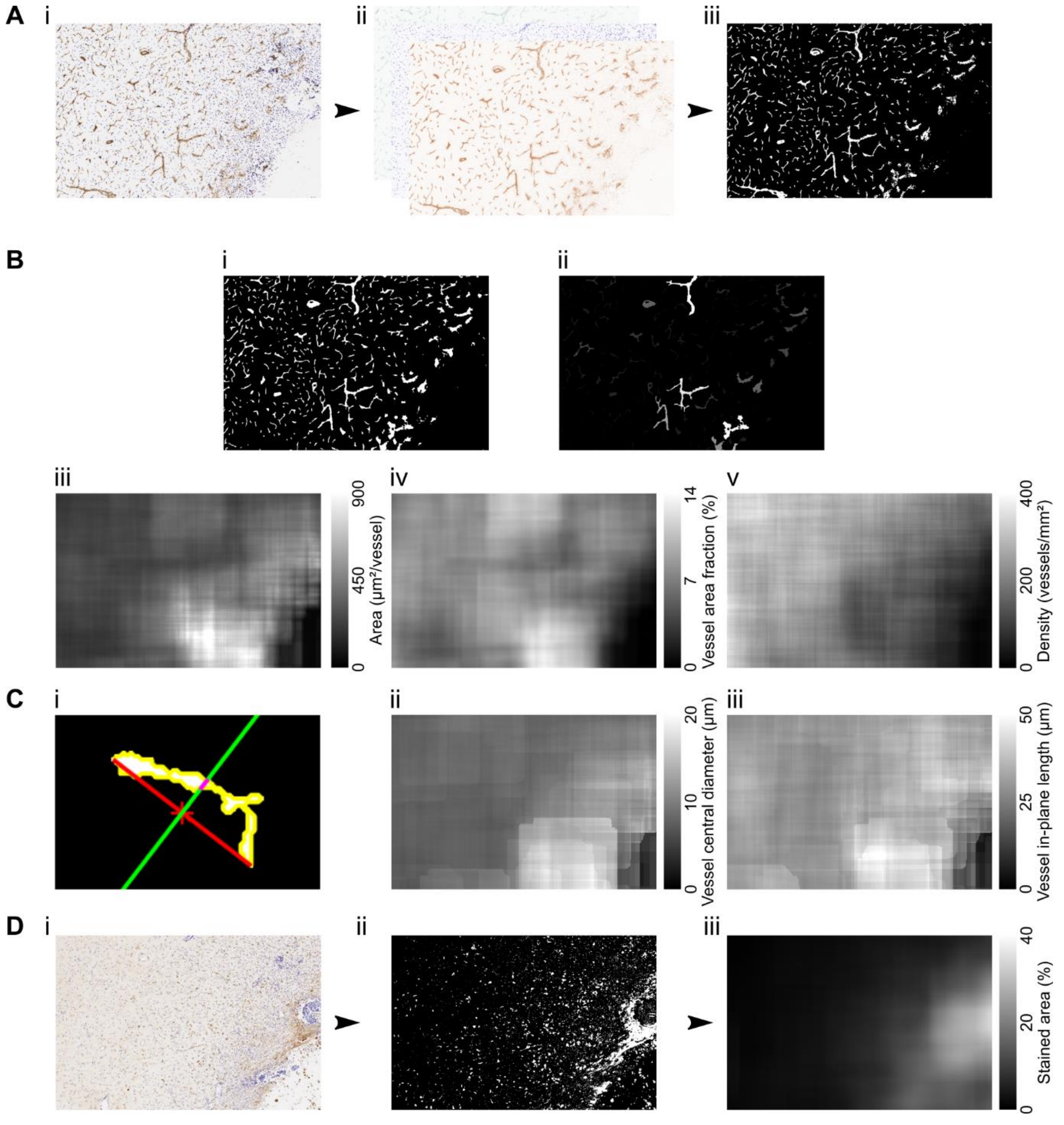

Figure E3: Example of histology analysis methods (A) Sections stained for collagen IV (i) were deconvolved to give just the brown component (ii) before being binary thresholded uniformly across all sections (iii). (B) Binary threshold masks (i) were processed via intermediary images (ii) to yield final maps of individual vessel cross-sectional area (iii), fraction of the area occupied by vessels (iv) and vessel density, irrespective of size (v). (C) (i) Each vessel (white) was isolated individually and processed to determine Feret diameter along long axis (red line) and vessel diameter at midpoint of long axis (pink section of green line). These parameters were used to calculate maps of (ii) vessel central diameter, and (iii) vessel in-plane length. (D) Pimonidazole staining of an equivalent section (i) was processed to yield binary masks (ii) and maps of stained area (iii).

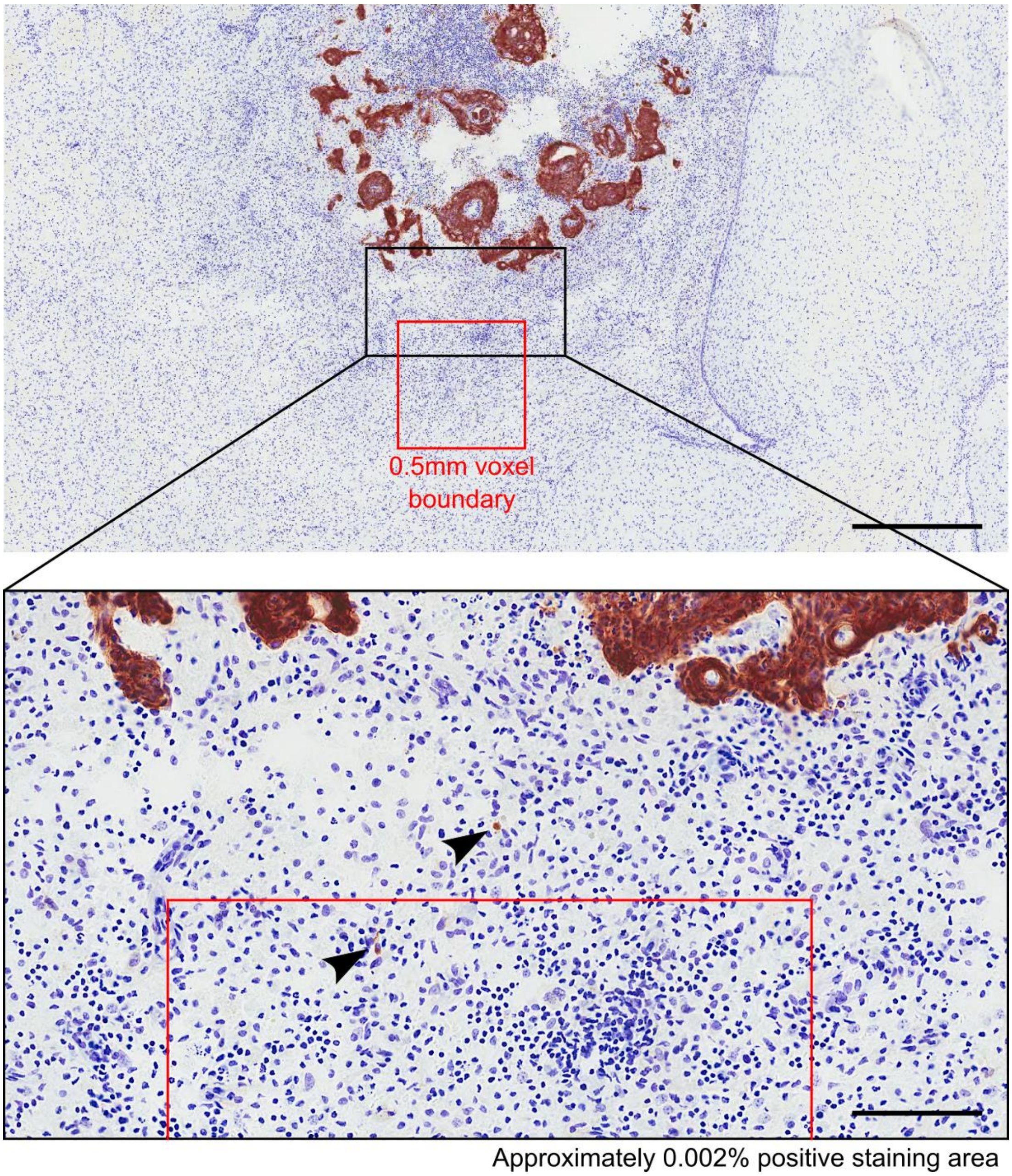

Figure E4: Determination of EpCAM staining threshold. EpCAM immunohistochemistry (brown) shows clear clusters of tumour cells, but also individual tumour cells that have migrated away from the main growth region (black arrows). In order to be an effective stain for identifying tumour, the voxel marked in red, identical in size to the MRI voxels, must be labelled as “positive” for tumour. This voxel is ~0.002% EpCAM positive, and so the threshold was set at 0.001% EpCAM positivity for “tumour-positive”. Scale bars: 500µm for widefield image and 100µm for magnified panel.

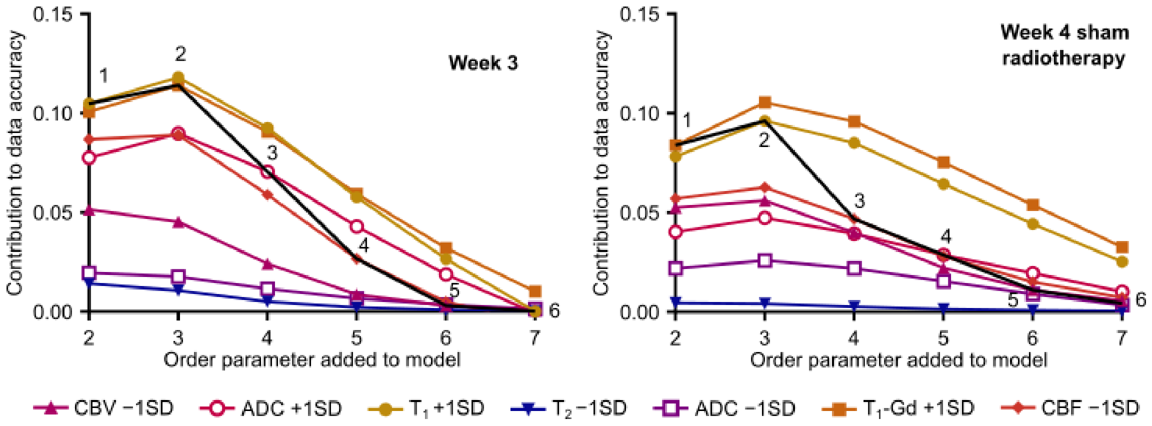

Figure E5: Contribution to data accuracy (delta accuracy) plot interpretation. Example illustrations showing a clinically useful interpretation of data accuracy contribution (delta accuracy) plots. The numbered black line proceeds from each addition point to the next one, each time intersecting with the highest delta accuracy modality that has not been chosen so far. By reading off the modalities in order, the optimal order of acquisition for maximum signal per unit time is revealed. e.g. for the Week 3 animals, the optimal order is T_1 +1SD, T_1-Gd +1SD, ADC +1SD, CBF -1SD, with ADC -1SD and CBV -1SD and T_2 -1SD all contributing minimal additional information.

Histological Property	Mean	Median	SD
Vessel area fraction (area of vessels/area of section)	8.3	8.3	2.6
Vessel central diameter (µm)	8.3	8.2	1.4
Vessel cross-sectional area (μm^2/vessel)	288	275	97
Vessel density (vessels/mm^2)	276	279	71
Vessel in-plane length (µm)	31	31	5
Cresyl violet positive area (%)	2.7	2.0†	2
Pimonidazole positive area (%)	0.1	0.0†	0.3

Table E1: Summary values for histologically-derived vessel parameters and staining densities for normal brain, derived from ROIs in the contralateral hemispheres of all 12 rats. † Median differs from mean by more than 5%.

Supplemental Methods E1

Animals and tumour induction

Female Berlin-Druckery (BD-IX) rats were housed in individually ventilated cages of 3 or 4, depending on body mass, under a 12h light/12h dark cycle with food and water ad libitum.

For tumour induction, rats (*n*=12, 150-180g) were anaesthetized with isoflurane (1.5-3% in 70:30 N_2O:O_2) and placed in a stereotactic frame. A burr-hole was made in the skull and 1000 ENU1564 cells were injected into the left striatum in 1μL PBS (coordinates: 1mm anterior and 3mm lateral to bregma, 3.5mm depth). The ENU1564 tumour cell line used in this study (kind gift from Professor G. Stoica, Texas A&M University) originated from an *N*-ethyl-*N*-nitrosourea-induced mammary adenocarcinoma in a female BD-IX rat, and is highly metastatic to brain and bone tissues. As a rat cell line, there is no publicly available short tandem repeat (STR) profile for the cells thus they have not been externally validated. Cells were tested for mycoplasma immediately prior to freezing each stock vial. Stock vials were revived, grown for up to one week in DMEM supplemented with 10% FBS and 1% 200mM glutamine, harvested by trypsinization, washed, then injected.

After recovery, animals followed the experimental flowchart given in Figure 1. Radiotherapy was delivered using a small animal radiation research platform (SARRP; Xstrahl, Camberley, UK). A single 25Gy fraction of 225kV X-rays was applied, with the tumour at isocentre of an 8mm diameter beam. The 8mm beam size was chosen to give sufficient margin to the largest tumour dimeter observable by post-Gd T_1-weighted MRI (6.4mm maximum diameter; mean diameter 5.4±0.9mm). Tumour isocentre was positioned by using T_1-weighted post-Gd MRI to determine isocentre relative to the skull midline, top of the cortex and the olfactory sulcus, before then transferring these relative coordinates to the CT of the head for irradiation (Figure E1). Prior to sacrifice, all animals received 60mg/kg pimonidazole HCl (Hypoxyprobe, Burlington, USA) i.p. in water. Brains were fixed in PLP_{light} (periodate-lysine-paraformaldehyde with 0.025% w/v glutaraldehyde) and cryosectioned for histology.

Histological processing

Animals were sacrificed by transcardial perfusion fixation using heparinized saline followed by PLP_{light} fixative (periodate-lysine-paraformaldehyde with 0.025% w/v glutaraldehyde). Brains were collected, post-fixed in PLP_{light} for 4-6h, cryopreserved in 30% (w/v) sucrose and frozen in OCT at -80°C before cryosectioning.

Brain sections (20μm thick) were stained to identify tumour cells (EpCAM), hypoxia (pimonidazole) or vessels (collagen IV). For EpCAM staining, sections were washed in PBS (5min, RT), incubated for 2x5 min in 300mL 10mM sodium citrate, pH 6.0, 750W microwave full power for antigen retrieval, washed with PBS, quenched with 1% (v/v) H_2O_2 (30% w/v) in methanol, blocked with 10% goat serum in PBS, incubated with primary antibody (rabbit anti-EpCAM; 1:2000, overnight, 4°C in PBS + 0.01% Tween-20, clone [EPR20533-266], Abcam, UK), rinsed in PBS, incubated with secondary biotinylated goat anti-rabbit antibody (1:500, 1h, RT, Vector Laboratories, Peterborough, UK), incubated with ABC development kit (Vector Laboratories) and developed with 3,3'-diaminobenzidine. Pimonidazole staining was identical except that no antigen retrieval step was used, the primary antibody was mouse anti-pimonidazole (clone 4.3.11.3, 1:100, overnight, 4°C, Hypoxyprobe) and the secondary antibody was horse anti-mouse (1:200, 1h, RT, Vector Laboratories). Collagen IV staining was identical except that initial washes were conducted at 37°C, antigen retrieval was in pepsin solution (1mg/mL in 0.2M HCl, 20min, 37°C), the primary antibody was an anti-rat collagen IV antibody (1:300 in 1% goat serum, 1h, room temperature (RT), ab6586, Abcam, UK), and the secondary antibody was a goat anti-rat

antibody (1:100, 1h, RT, Vector Laboratories). All stained slides were counter-stained with cresyl violet, mounted and digitally scanned at 200x magnification using an Aperio ScanScope slide scanner (Leica Biosystems, Milton Keynes, UK).

Magnetic resonance imaging acquisition details

All MRI was conducted in a 9.4T horizontal-bore scanner (Agilent Technologies Inc., Santa Clara, USA) with a 72mm volume transmit coil and a 4-channel surface-receive array (Rapid Biomedical, Rimpar, Germany). Quantitative map parameters: T_1 map by inversion-recovery, TI=13-8000ms, TR=10s; T_2 map by multi-echo acquisition, TE=30-160ms, TR=10s; CBF maps: multiphase pseudo-continuous arterial spin labelling[1]; CBV maps from T_2 maps acquired before and after intravenous injection of ultra-small particles of iron oxide (USPIO, 30nm diameter, 10.6mg Fe/mL, 100μL/rat, relaxivity 1820Hz/(mg/mL) at 9.4T, synthesized in-house[2]); ADC maps from diffusion-weighted images acquired in three orthogonal directions for b=0s/mm² and b=1000s/mm². All data were acquired using a spin-echo echo planar imaging (EPI) readout; FOV=32x32mm, matrix=64x64, thickness=1mm, 10 slices.

Parameters for T_1-weighted images (both before and after administration of 150μL gadodiamide via an indwelling tail vein cannula) and T_2-weighted images: FOV=32x32mm, matrix=256x256, thickness=1mm, 10 slices; T_1: TR=500ms, TE=20ms; T_2: TR=3000ms, TE_{eff}=51.3ms. The high-resolution T_1-weighted images were down-scaled to matrix=64x64 with the same FOV to create low-resolution images for tumour masking comparisons.

Tumour extent maps

Histological tumour extent maps were produced from perspective-transformed MRI-resolution histological datasets. Histological sections (20μm thick) were taken every 200μm, meaning that five sections covered the thickness of one MRI slice (1mm). Perspective transform matrices were manually determined for each slice (using overlays in the GNU Image Manipulation Program v2.8) to account for distortions of the tissue owing to either shrinkage during processing or damage during sectioning (Figure E2). Parameter maps were produced (Figure E3) from each histological section and perspective transform matrices were applied using Matlab (in-house scripts, code available on request). Histological tumour extent was carried out at MRI resolution, in MRI-space, after transformation and combination of all histological information. This process meant that not only could histological volume be determined but that also spatial overlap values could be calculated.

For human determination of tumour volume, four independent observers (JRL, MAS, AdB, VAJ) each delineated maximum tumour extent on low-resolution then high-resolution post-Gd T_1-weighted images (voxels were 0.5mm² and 0.125mm² in-plane, respectively; 1mm slice thickness). Observers worked on all low-resolution images first then proceeded to high-resolution images to avoid bias arising from alternating high then low resolution images. A single consensus independent observer tumour map was produced by summing the maps from the four observers then excluding voxels where only a single observer thought there was a tumour present.

To assess whether simple automated analysis of the T_1-weighted post-Gd images offered improvement over the human observers, two methods of identifying tumours from post-Gd T_1-weighted images were used: (1) voxels >2SD above the mean signal intensity of the contralateral (normal) hemisphere; and (2) voxels defined by an automated routine designed to more accurately emulate steps a human observer intuitively makes (termed the "refined" method,) (i) begin with the mask of voxels >2SD above the contralateral mean, (ii) for each slice eliminate regions that are single voxels, not connected to any other

voxels, (iii) for each slice smooth the shape by dilating then eroding each region by two voxels, (iv) keep only the largest contiguous (26-connected) region, and (v) fill any interior holes in this region.

Justification for MRI abnormality threshold

Multimodality MRI-based tumour maps were produced by considering voxels to be tumour if two or more MRI-based parameters were abnormal. The two-parameter threshold was chosen to minimize false positives by keeping the significance level (α) <0.05. For a one-sided test combining *n* normally distributed parameters each *Z* standard deviations from the mean, α is given by:

$$\alpha = \left(1 - \left(\frac{1}{2}\left[1 + \operatorname{erf}\left(\frac{Z-\mu}{\sigma\sqrt{2}}\right)\right]\right)\right)^n$$

where μ=0, and σ=1 for the standard normal distribution. With a threshold of one SD from the mean (i.e. Z=1), α=0.16 for n=1 which is insufficient to satisfy a criterion of α<0.05. However, combining two modalities (n=2, both Z=1) gives α=0.025 which satisfies the condition of α<0.05. Therefore, combining two modalities, each 1SD from the mean, gives sufficient confidence that false positive voxels will be minimized.

Predictions, model analysis, and modality importance

Model accuracy for tumour identification was determined using $Accuracy = (TP + TN)/(TP + FP + TN + FN)$ where TP is True Positive, TN is True Negative, FP is False Positive and FN is False Negative. Classification models were constructed with all possible permutations and combinations of MRI modalities included. By inspecting the model accuracies before and after each modality addition, (i) relative contributions to overall model accuracy for each modality were determined, and (ii) the most beneficial order of combination of modalities was determined.

The extent of overlap of tumour areas between observers was calculated using the Jaccard index (J), where $J_{(Observer\ A, Observer\ B)} = |A \cap B|/|A \cup B|$.

To investigate which MRI modalities were most important for finding clinically occult tumour, each brain was processed to remove voxels identified as tumour by independent observers. The resultant brains were assessed by multimodal MRI to locate residual tumour and the modalities contributing the most accuracy to identification of tumour were determined.

Supplemental results

Agreements between independent observers for tumour delineation

Delineated tumour volume was not significantly different between high and low-resolution MR images when considering all four observers as a group. Pearson's correlation coefficient between all pairs of observers was 0.94±0.03 and 0.96±0.02 for low and high-resolution images, respectively (mean±SD). Intra-brain, inter-observer error in overall tumour volume was 16±8% and 14±7% for low and high-resolution images, respectively.

Although delineated tumour volume was not significantly different between high and low-resolution MR images when considering all four observers as a group, individual observers did exhibit differences in delineated tumour volume: one observer drew larger tumours and two observers drew smaller tumours on the high-resolution images compared to the low-resolution images. One observer drew identically-sized tumours at both resolutions.

Independent observer agreement was similar to previously published multi-centre analyses[3]. Intra-brain, inter-observer error in overall delineated tumour volume was 14±7% on high-resolution images. For tumour-volume overlap, J_{mean} between all pairs of observers for all tumours was significantly higher with the high-resolution images than with the low-resolution images (0.76±0.06 and 0.73±0.06, respectively; $p<0.05$ Wilcoxon signed-rank test). J_{mean} within observer and between resolutions was 0.70±0.06, significantly lower than between observers at either single resolution ($p<0.05$, Wilcoxon signed-rank test). Mean inter-observer variation in J for all tumours was lower for high-resolution images (6.1±4%) than low-resolution images (8.9±6%; $p<0.05$, Wilcoxon signed-rank test).

Within the population of voxels identified as tumour in an animal by at least one observer, the majority (72±8%) were classified as tumour by all four observers. Overall, 89±9% of voxels were identified as tumour by at least 2 observers, with only 11±5% being identified as tumour by a single observer (Figure E6). Voxels classified as tumour by only a single observer were predominantly found on the periphery of the delineated tumour volumes.

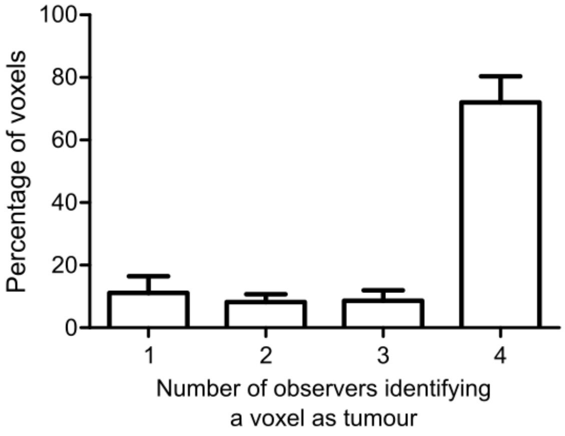

Figure E6: Agreement between independent observers. Number of observers (out of four) that agreed that a particular voxel was tumour.

Addition of different number of modalities to identify tumour using MRI

The optimum number of MRI modalities to sum to identify a voxel as being tumour-positive was two. Results did not improve if this threshold was increased to three or more modalities. This result is related to the calculation of α (the likelihood that the true population parameter lies outside the confidence interval).

Over the first five modality additions, α is:

- 1 modality: $\alpha = 0.158$
- 2 modalities: $\alpha = 0.025$
- 3 modalities: $\alpha = 0.004$
- 4 modalities: $\alpha = 0.0006$
- 5 modalities: $\alpha = 0.0001$

One modality is insufficient to satisfy the criterion that $\alpha<0.05$ and was thus prone to false positives. Modality numbers higher than this are all sufficient to meet this criterion, but become increasingly stringent, i.e. α becomes smaller. Consequently, as the number of modalities required to identify tumour

increased, specificity increased, but sensitivity decreased. However, since specificity and sensitivity were already both high, the consequence was that specificity rapidly approached 1, whilst sensitivity dropped to 0. Overall model accuracy ($Accuracy = [TP + TN] / [TP + TN + FP + FN]$), which took these into account, peaked with two modalities identifying tumour, and decreased at higher modality numbers.

References

1. Larkin JR, Simard MA, Khrapitchev AA, et al. Quantitative blood flow measurement in rat brain with multiphase arterial spin labelling magnetic resonance imaging. *J Cereb Blood Flow Metab*. January 2018:271678X18756218.

2. Perez-Balderas F, Kasteren SI van, Aljabali AAA, et al. Covalent assembly of nanoparticles as a peptidase-degradable platform for molecular MRI. *Nature Communications*. 2017;8:14254.

3. Sandström H, Jokura H, Chung C, Toma-Dasu I. Multi-institutional study of the variability in target delineation for six targets commonly treated with radiosurgery. *Acta Oncologica*. 2018;57(11):1515-1520.
